# New Publications

**Published:** 1899-02

**Authors:** 


### NEW PUBLICATIONS.

PRACTICE OF EQUINE MEDICINE: A Manual for Sudents and Practitioners of Veterinary Medicine. Arranged with Questions and Answers. With an Appendix containing Prescriptions for the Horse and the Dog. By HARRY D. HANSON, D.V.S. New York: H. D. Hanson & Brother, 1899.

THIS little work of 259 pages is, as its title indicates, a review of the practice of equine medicine for the use of students and practitioners. It is arranged in the form of questions and answers, so that ready reference may be made to any part of the subject that the reader wishes to inform himself upon. The scope of the work may well be indicated by giving the titles of the chapters or sections. These are as follows: Introduction, including general and special pathology; The Classification and Causes of Diseases; Terminations of Disease and Modes of Death; Inflammation, including atrophy and degeneration; Classification of Diseases, including fevers, diseases or changes in the blood, infectious and epizoötic diseases, and constitutional diseases; Diseases of the Respiratory System, including physical diagnosis and the diseases of the different parts of this system; Diseases of the Digestive System; Diseases of the Urinary System; Diseases of the Circulatory System; Diseases of the Diaphragm; Diseases of the Nervous System, and, lastly, Intoxications.

The appendix contains a number of tried and approved prescriptions used in equine and canine practice.

The purpose of the book, as expressed in the preface, is to provide a condensed work on the theory and practice of equine pathology. It is intended to serve as a help to students in getting their degrees and in passing State Boards, and to practitioners for quick reference.

The English and American authors of text-books have been drawn upon in obtaining the material for the work. Much of the advice furnished is deduced from the results of the author's own extensive experience. The sections on Treatment are especially complete, although diagnosis has received much attention.

The book is very well printed, on good paper, and is furnished with a copious index, so that any part of any subject may be referred to without loss of time. The work is well adapted to the purpose for which it is intended.

Concentrated Feed-stuffs. Bulletin No. 56 of the Hatch Experiment Station of the Massachusetts Agricultural College.

This bulletin gives an excellent report of the relative feeding value of the many prepared animal and poultry foods on the market. The investigation of the merits of these commercial products has been from a practical point of view, and their measure of worth in dollars and cents quickly obtained by the intelligent stock-owner. The adulterations of many of the animal food-stuffs are pointed out, and much of value is to be learned by veterinarians in these bulletins that tends to complete the education of the members of the profession in zoötechnics, of so much practical importance to-day in the demand for economy in maintenance and output of animal industry.

Prescription Writing. By Prof. H. D. Hanson.

This work is in preparation, but, owing to some delinquents who had kindly promised prescriptions for the appendix and who have neglected thus far to send them, the work has been delayed. The author has about one-half the required number, and hopes to secure the remainder shortly.

---

Dr. W. N. D. Bird, recently stationed at Arkansas City, Kansas, has been transferred to Nashville, Tenn., for inspection work along the Texas-fever quarantine line.

Dr. C. S. McKenna, of Washington, Pa., is now a sojourner in Denver, Colorado, in search of restoration of health.

Dr. Cabot, of New York City, read a paper on "Rabies" at the Academy of Medicine of that city on February 21st. Several prominent veterinarians discussed the subject.

Dr. Leonard Pearson, State Veterinarian, was a visitor to Pittsburg in January, subsequent to the reorganization of the State Live-stock Sanitary Board, the newly elected Governor, Wm. A. Stone, having been elected President in accordance with the provisions of the Act.

Drs. John W. Adams, B. M. Underhill, George Jobson, J. C. McNeil, C. J. Marshall, W. L. Rhoads, W. H. Ridge, and W. Horace Hoskins were a committee of veterinarians representing the Pennsylvania and Keystone Veterinary Medical Associations who waited upon Governor Stone on February 1st to urge the retention and reappointment of Dr. Leonard Pearson as State Veterinarian of Pennsylvania.

